# Pathways to a diagnosis of trigeminal neuralgia: a qualitative study of patients’ experiences

**DOI:** 10.1186/s12875-025-02763-8

**Published:** 2025-03-06

**Authors:** Cameron Werner, Jeni Harden, Julia Lawton

**Affiliations:** 1 Department of Psychology, IU International University of Applied Sciences, Erfurt, Germany; 2 Usher Institute, Medical School, University of Edinburgh, Edinburgh, UK

**Keywords:** Trigeminal neuralgia, Qualitative research, Diagnosis, Patient experience

## Abstract

**Background:**

Trigeminal Neuralgia (TN) is a rare disorder which causes episodes of intense facial pain and has been described as the ‘suicide disease’. Hence, prompt diagnosis and timely initiation of treatment is vital. However, delays to diagnosis and high rates of misdiagnosis are common, particularly within primary care. To date, most research has focused upon treatment options rather than improving diagnostic experiences. This study sought to explore patients’ experiences of the events leading up to their TN diagnosis and their views about the care and support they received when they were diagnosed to provide recommendations for improving the TN diagnostic pathway.

**Methods:**

This was a qualitative, exploratory study using in-depth interviews. Interviews were conducted with (*n* = 25) UK-based people with TN recruited via online forums. Data were analysed thematically.

**Results:**

Following the onset of their TN pain, most participants described an arduous and uncertain journey to diagnosis, with many encountering significant delays, misdiagnoses and receiving inappropriate referrals and treatment. As a consequence, participants reported experiencing profound distress, anxiety, depression and, in extreme cases, suicidal ideation; some also described drug and alcohol misuse during this time. Most participants conveyed relief upon finally receiving a diagnosis. However, this was often by eclipsed by what they saw as poor and insensitive communication and inadequate information provisioning.

**Conclusions:**

The present study highlights the importance of developing bespoke training for primary care and other professionals to facilitate timely recognition of TN symptomatology and ensure that they deliver a TN diagnosis in clear, sensitive and empathetic ways.

## Background

Trigeminal neuralgia (TN) is a rare form of orofacial neuropathic pain in one or more divisions of the trigeminal nerve. The condition is characterised by recurrent, sudden episodes of severe pain to the face, typically described as shooting or stabbing [1]. TN attacks may be triggered by activities such as eating, speaking and touch [2–4]. The condition has been described as one of the most excruciating disorders known, often causing considerable disruption and distress to those affected [3, 4]. Individuals with TN may exhibit considerable anxiety engaging in triggering activities and go to great lengths to avoid these. In severe cases, individuals may experience malnutrition, dehydration [3, 5–7] and suicidality [4]. Given the negative clinical and quality-of-life impacts, and the lack of response to traditional analgesics, prompt diagnosis is vital. However, underdiagnosis, delays to diagnosis and high rates of misdiagnosis are common, particularly within primary care settings [8–11].

To date, most TN research has focussed upon treatment options [12–17] rather than improving the diagnostic pathway. While qualitative research can help ensure that healthcare services are responsive to patients’ needs [18, 19], limited qualitative research has explored the perspectives of people with TN [20, 21] and no study has focused specifically on their experiences of a TN diagnosis. Healthcare professionals may be able to improve the speed and accuracy of diagnosis by using insights gained from patients’ perspectives [22]. This study sought to explore patients’ experiences of the events leading up to their TN diagnosis and their views about the care and support they received when they were diagnosed. The objective was to provide recommendations to help improve patients’ diagnostic experiences.

## Methods

As the study aimed to understand patients' lived experience of TN, in-depth interviews, informed by topic guides, were used, as they allowed individual experiences to be captured in detail, while simultaneously allowing participants opportunities to discuss issues which really mattered to them, including those unforeseen at the study outset [23]. The study design was also informed by the first author’s lived experience of TN and consultation with other individuals who had TN. In light of this initial consultation work with non-study participants, it was recognised that asking individuals to travel for a research interview could pose an insurmountable hurdle, particularly if they were currently experiencing severe pain. Hence, a decision was made to use telephone interviews which have previously been described as a practical and naturalistic method for collecting data from individuals in hard-to-reach populations [24]. Participants for whom speaking could act as a pain trigger were also offered opportunities to take several shorter interviews or to be interviewed via email. Participants were also advised that they could terminate their interview at any time, should they encounter pain simply by hanging up. They were also advised that, in the event that they experienced upset or distress during their interview, they would be given contact details of a TN support group.

### Participants and sampling

Participants were recruited through an online advertisement placed upon the Trigeminal Neuralgia Association (TNA – the largest UK-based charity dedicated to TN) website and a large, closed forum for individuals with TN and an opt-in approach was used. Purposive sampling was used to attain a sample which broadly reflected the wider TN population in terms of age gender, occupation and educational attainment. Because TN disproportionately affects women [9, 10] we sought to recruit a larger number of women than men. Recruitment continued until data of sufficient depth and breadth had been collected to address the study aims.

### Data collection

Interview guides were developed in light of literature reviews, discussions with the TNA and consultation with individuals with TN (who were not study participants) and revised in light of emerging findings (see Appendix 1 for details of the main areas covered). All interviews were undertaken by the first author (CW). Interviews lasted 48—149 min, with seven participants opting to take part in 2–3 shorter interviews due to pain levels. All interviews and were digitally recorded and transcribed in full.

### Data analysis

To maximise rigor and reduce risk of researcher bias all three team members were involved in data analysis. In line with an inductive approach, data analysis commenced as soon as data collection began, allowing findings emerging from earlier interviews to iteratively inform areas explored in later ones. The process of reviewing transcripts and iteratively revising the topic guide continued until team members agreed that the areas explored allowed all of the information to be captured which was needed to address the study aims. The final analysis used both inductive and deductive thematic approaches. Individual interviews were read through repeatedly (data immersion) before being cross-compared to identify overarching themes, with CW leading the analysis, and JL and JH (both highly experienced qualitative researchers) supporting identification and validation of themes. Data were coded using these overarching themes and coded datasets where subject to further analysis to identify subthemes. NVivo 10 (QSR International Pty Ltd., Doncaster, Australia) was used to facilitate data coding and retrieval.

### Ethical issues

Ethical approval for the study was granted by Usher Research Ethics Committee (reference number 1765). All participants provided written informed consent prior to data collection. To safeguard participants’ anonymity, pseudonyms are used in the reporting below. To further help to protect their identities, and after it became apparent during some interviews that some participants knew each other via the online forums, we also decided not to include information about their current/previous occupations in this article.

## Results

The final sample comprised *n* = 25 individuals whose ages ranged from 29 – 83 years. For further information about the sample, see Table 1.

**Table 1:** Sample characteristics

Characteristic	n	%
**Sex**		
Male	6	24%
Female	19	76%
**Educational attainment**		
No qualification / NVQ	1	4%
GCSE / O level	3	12%
A levels or equivalent	3	12%
Tertiary (Undergraduate degree)	8	32%
Tertiary (Postgraduate degree)	8	32%
Declined to say	2	8%
**Ethnicity**		
White British	23	92%
Mixed Caribbean	1	4%
White & Asian	1	4%

For most individuals, the beginnings of their pain heralded the advent of an often arduous and uncertain trajectory to diagnosis, with many encountering significant delays, ranging from several weeks to many years. During this time, participants reported experiencing profound distress and uncertainty, with many also describing the considerable effort they had to go to to be taken seriously by healthcare practitioners and given the correct diagnosis. Below, these experiences are considered in more detail together with the negative psychological, emotional and clinical impacts resulting from a delayed TN diagnosis and subsequent poor communication and information delivery.

### Initial symptoms as the beginning of a journey

Many participants described the onset of their pain as severe, sudden and often extremely debilitating and anxiety-provoking:

*“It’s like a bolt of lightning inside your head, really sharp, really sudden (...) I just remember having sharp shooting pains…And I thought ‘something’s not right, I don’t know what it is’. And it made me go to the out-of-hours place because it was excruciating.” (Hermione)*

In some cases, such as Hermione’s, the severity and rapid onset of their pain prompted participants to seek urgent medical assistance by presenting at out-of-hours doctors’ services and emergency departments or making use of urgent appointments with general or dental practitioners due to TN’s propensity to mimic facial or dental pain.

Other participants reported a more insidious onset to their TN wherein they had initially experienced less intense pain which became increasingly severe over time. Such participants described struggling to make sense of their pain which could disappear and reappear over the course of days, weeks, months or even years and, consequently, contemplating a wide range of potential causes including: sinus infections, gum recession, allergic reactions, viruses, toothache and complications arising from earlier dental work. As a result of these experiences and understandings, several reported seeking medical consultations from general practitioners and dentists, albeit not with the same urgency as those described above:

*“It felt as though it could have been toothache because I didn’t know any different at the time. (...) I think my first bits of pain weren’t as severe, as it did develop. It felt like when you’ve been at the dentist and the dentist, when they’re giving you an injection and they just hit a nerve. It felt a bit like that. And I suppose because I had nothing to compare it with, I was thinking dental myself.” (Rita)*

Others, however, reported putting off seeking medical assistance due to concerns that their pain would not be taken seriously*.* Because of the condition’s tendency to go into remission spontaneously, some also assumed their issue had resolved itself and, hence, that medical input was no longer needed.

### Navigating misdiagnosis and advocating for further testing

While a small number of participants received an immediate diagnosis after presenting to healthcare professionals, the majority reported experiencing considerable delays. In many cases, participants described initially receiving unsatisfactory explanations for their pain, most typically from a GP or dentist. Often these explanations, particularly those offered early in the diagnostic journey, were perceived as trivialising or being dismissive of their symptoms and the level of pain and distress they were in:

*“I went to the dentist, local dentist and she said ‘oh yeah, you’ve got a little bit of bacteria’ (…) and I said ‘I literally, I can’t move’. I couldn’t eat, couldn’t speak, I could barely put water in my mouth to drink water. And she said, she kind of brushed it off and said ‘oh there’s nothing wrong with you’ (...) the dentist actually said ‘your teeth are absolutely fine, they’re solid’ (...) she kind of said to me ‘I don’t even know why you’re crying when it’s that tiny little bit of decay’. And that made me feel a bit like, ‘mm, thanks a lot, I’ve been through childbirth and you’ve said that to me.” (Molly)*

As well as feeling angry, rejected and delegitimised as a result of these kinds of medical encounters, participants reported needing to actively resist healthcare professionals’ explanations and push for further tests, scans or referrals themselves.

*“I thought I had chronic toothache (…) I went to the dentist- I got an emergency appointment. He didn’t say anything about TN to me at all, he just told me I’d got perfectly good teeth and I demanded an x-ray because I said I’m in agony, there’s something wrong.” (Lavender)*

Given the ambiguous nature of their pain, however, participants reported uncertainty as to which specialists they should try to seek help from. Participants also noted how this ambiguity around the nature of their symptoms resulted in medical uncertainty, with several being referred back and forth between medical professionals who did not consider their symptoms to be within their remit:

*“It got to the point where there was no point going to A&E because they weren’t doing anything. So (…) I spoke to my GP about what kind of consultants I should see and that’s when I saw the ENT. And the ENT said maybe you should see the neurologist. And because after the MRI was clear the neurologist said ‘see the maxillofacial’ and then I saw the maxillofacial and but they just said ‘see the ENT’, so I just got sent round in circles a bit.” (Luna)*

Some participants also reflected on their own role in their delayed diagnosis, by highlighting the challenges they encountered communicating their pain to their medical practitioners. Many spoke of TN pain as a unique form of pain, unlike anything they had previously encountered and, consequently, feeling unable to put their suffering into words.

*“No one else gets it. It’s like you can’t explain to someone (…) up until I got this, I’ve never had anything like it (…) It’s just this earth-stopping pain.” (Lily)*

Other participants reported not only experiencing difficulties relaying the nature of their pain but also struggling to pinpoint its exact location, which could further hamper their ability to receive the correct diagnosis and receive effective pain relief:

*“I ended up going to the doctor’s [GP] and (...) she thought I had something called temporomandibular jaw disorder [sic] (...) And my pain was emanating from my upper jaw (...) so I accepted her diagnosis and she gave me codeine and tramadol and more or less said just eat sloppy food and off you go, type of thing. So, because I’m too polite and I accepted the diagnosis (...) but my language was too polite about the intensity of the pain I was in. I was saying things like ‘I’m finding it very difficult to manage it’ instead of saying ‘this is like nothing else I’ve ever experienced in my life, you need to help me!” (Poppy).*

### Tipping points to diagnosis

As a result of feeling dismissed, not taken seriously, or dissatisfied with the treatment and care they had received, some participants reported undertaking their own independent research. In doing so, several noted how they had actively contributed to their eventual diagnosis. This included Lily who had presented to her GP on a number of occasions, but only succeeded in getting a TN diagnosis after sharing a newspaper article with them:

*“I am ashamed to say that I was googling and I came across, of all articles, a Daily Mail one. And like most scientists I hate the Daily Mail however I can kind of put my diagnosis down to them really. I saw this article which was called ‘The toothache that is nothing to do with your teeth (’…) and I read that and (…) I just went ‘oh my god, that’s it!’ (…) So I went to my GP and I said ‘I think I’ve got trigeminal neuralgia, what do you think?’ and she agreed.” (Lily)*

For others, however, it was only after a crisis point had been reached that an eventual diagnosis was made. Such participants reported severe incapacitation due to experiencing excruciating levels of pain, with their situations having become so extreme that their distress and suffering was overt and unmistakable to GPs:

*“I wonder if people say they don’t believe you when you say you’re in pain. Because as I was trying to explain, they just sent me all over the place. I think they only took real notice of me when they saw me covered in sick, screaming and crying (…) And because he’d [GP] seen me like that, then he believed me I guess.” (Charlie)*

Indeed, in some cases, such as Penelope’s below, participants reported how it had taken an emotional breakdown in front of a healthcare provider to catalyse their diagnosis. Penelope had previously experienced considerable delays as a result of being referred back and forth between her GP and dentist, adding to her desperation at being made to wait further despite her agonising pain:

*“So if he said ’come back this afternoon, I’ll do it this afternoon’ I would have gone and done it but when he said come back in two weeks I sort of said ‘OH GOD, OH NO NO NO NO’ and I reacted quite violently and (…) then he must have thought ‘this isn’t right, there’s something wrong here and he said “I’ve got a colleague who works in the dental department of (names hospital), let me consult him.” (Penelope)*

### Psychological, emotional and clinical impacts of a delayed diagnosis

Experiencing delays to their diagnosis often appeared to have significant negative psychological and emotional impacts due participants feeling belittled and dismissed by healthcare professionals: “it felt *like I was going crazy*” as Luna put it, or as Susan described: “*I just felt very alone.”* In extreme cases, feelings of hopelessness, isolation and despair, coupled with inadequate pain control, resulted in suicidal ideation:

*“When he [GP] said come back in two weeks I said ‘I can’t go through any more’ it had already been two weeks that I had been suffering and I really couldn’t stand any more pain. It was quite unbearable, it was really making me demented, I mean I was becoming, you know, I was seriously suicidal, I was thinking there would be no other way to end the pain than to kill myself.” (Penelope)*

Given that many participants were provided with limited, if any, effective pain relief by their GPs, some reported trying to treat their pain at home. In their endeavours to do so, participants discussed resorting to potentially dangerous behaviours; such as taking significant amounts of off-the-shelf painkillers concurrently with alcohol to strengthen their effects. Furthermore, as a result of seeing multiple, often inappropriate, healthcare professionals during their diagnostic journey, many also described receiving a range of misdiagnoses. This, as participants further noted, often resulted in them undergoing unnecessary and sometimes invasive medical procedures ranging from administration of antibiotics and removal of fillings, to more harmful and risky procedures, such as root canal surgery and unwarranted dental extractions:

*“It started in May 2016 and the first time that trigeminal neuralgia got mentioned was October 2017 (...) and that was after I’d had a load of teeth pulled out. (...) I had the four top left teeth pulled out and I had three lower left teeth pulled out (…) Well, I was demanding them out, I was in agony with them (…) I was just so convinced that it was my teeth.” (Luna)*

### Experiences of diagnosis

Due to seeking assistance from a variety of medical practitioners, participants’ TN diagnoses were provided by a diversity of medical practitioners, including GPs, dentists, maxillofacial surgeons, ENT specialists and neurologists. For some, receiving a TN diagnosis was described as a relief, because it gave legitimacy to their suffering and allowed them access to treatment. However, most reported negative experiences resulting from being given very limited information and the perceived lack of acknowledgement of how the diagnosis affected them:

*“I felt like I've been given a death sentence. I couldn't understand how a doctor could, (…) after all I've been through, give me ten minutes, shove this piece of paper in my hand, give me a thingy for carbamazepine and shoo me out the door and said ‘see you in a month.” (Lavender)*

Several participants also reporting experiencing extreme distress upon realising that their pain resulted from a chronic condition and, hence, that there was no quick or easy fix:

*“I was pretty devastated because suddenly it went from this might be something I can fix easily, one of my teeth if I can find out which is doing it, to no this is it, I will have this forever.” (Lily)*

Participants also reported struggling to assimilate the information given at the time of diagnosis due to being in extreme pain, distressed, anxious and sleep deprived and healthcare professionals using excessive medical terminology. As a consequence, some conveyed regret they had not had a partner/family member present at the time:

*“I was in such a state I could not even remember the instructions he gave me about how to gently increase the dosage you know? And I couldn't because I was sleep deprived, I was absolutely physically and mentally exhausted and I was in excruciating pain (…) I wasn't functioning. My husband unfortunately wasn't here.” (Lavender)*

As a consequence, participants often described independently accessing information about TN e.g., through online searches. While some described this as empowering and allowing them to better engage with healthcare professionals, others highlighted the highly detrimental impact of discovering that TN lacked a cure, treatments could have limited efficacy, and that the condition had been labelled the ‘suicide disease’:

*“I went straight on to PubMed and I found out about TN. When I started reading I thought ‘oh no, the suicide disease, what on earth is all this about?’ and then I realised the future was going to be (…) quite grim. And that the treatments were severe, pretty major (…) So I was pretty desperate.” (Ron)*

## Discussion

This qualitative study has explored in-depth patients’ experiences of being diagnosed with TN. As we have shown, many individuals presenting with TN pain experienced protracted journeys to diagnosis, which, as also noted in the literature [4, 8–11], often involved multiple clinical encounters and misdiagnoses. Consistent with the wider literature [4, 25–27], our findings have also highlighted a plethora of negative psychological consequences that can arise from a delayed TN diagnosis, including anxiety, distress, depression and, in extreme cases, suicidal ideation. In addition, we have shown how, as a result of diagnostic errors, patients may be subjected to invasive and unnecessary medical interventions, such as unwarranted dental extractions and use of antibiotics. While exposure to unnecessary medical interventions has previously been reported [8, 28] our findings highlight the ‘real world’ impacts these can have. Taken together, our findings underscore the importance of the diagnostic pathway being improved, particularly given the risk of suicide [4] and drug/alcohol misuse if TN remains undiagnosed.

As others have argued, it is important to use patients’ experiences to encourage and inform swifter TN diagnoses [22]. It has also been noted that, in the absence of accurate diagnostic testing, diagnosing TN remains complex and challenging [29] as it needs to be made on the basis of a patient’s personal history and description of symptoms [11, 30, 31]. Given the difficulties our participants reported conveying the nature and intensity of their pain, and TN’s rarity which may mean that primary care practitioners only encounter several cases during their careers [32], it is unsurprising that underdiagnosis and misdiagnoses by GPs and others are so common [8–11]. Hence, as others have argued, it is vital that training of primary care and other (e.g., dental) professionals be improved to raise their awareness of TN and improve the speed of diagnosis [33, 34]. As part of this training, GPs and other healthcare professionals would benefit from being made aware of the difficulties patients encounter verbalising TN pain [16], and taught ways to recognise verbal and non-verbal signifiers of the disease, such as twitching and grimacing (facial) responses to pain [35, 36]. In a UK context, it is also vital that the most up-to-date NICE and Royal College of Surgeons clinical guidance [37, 38] is disseminated widely to primary care and other relevant healthcare professionals; similar guidelines should be widely distributed in other countries where they are available. Additionally, consideration could be given to population-level TN awareness-raising campaigns. However, if this option is pursued, careful thought would need to be paid to how to ameliorate risk of distress in cases where individuals discover they likely have TN (the ‘suicide disease’) without appropriate emotional and/or psychological support being in place.

While many participants conveyed relief at finally having an explanation for their pain, this was often eclipsed by what they saw as poor and insensitive communication. From healthcare professionals’ perspectives, a TN diagnosis may not be considered serious as the condition is not life-threatening. However, as our participants’ accounts powerfully highlight, being told one has TN can cause considerable distress. Hence, it is vital that clinicians proceed sensitively and carefully in consultations and acknowledge the ‘human significance’ of a TN diagnosis [39]. In some cases, as our findings further indicate, referral to a psychologist or counsellor may be appropriate.

Our findings also suggest that individuals require (more) comprehensive information following a TN diagnosis. Indeed, participants indicated that being given a brief information leaflet at diagnosis was not only inadequate but also made them feel belittled, unsupported, and that the person delivering the diagnosis had failed to acknowledge the gravity of their news. Additionally, many reported how the brevity of their diagnostic encounter had foreclosed their ability to ask questions and gain more information and understanding of TN and its implications. Clinicians should therefore be aware of the need for more detailed information, including written information about dosing regimens given participants’ difficulties assimilating details of TN management at the time of diagnosis. Clinicians could also consider offering patients a follow-up appointment shortly after the diagnosis, to give them opportunities to ask questions and seek further information about TN when they might better placed emotionally to assimilate it. It is also important that all information relayed during such consultations is provided in clear, everyday language, and that overly medicalised terminology is avoided. In light of our own findings, it may also be helpful to encourage patients to consider bringing a partner or family member along to appointments where a TN diagnosis and its implications are discussed.

### Strengths and limitations

This study has provided novel insights into TN patients’ experiences prior to and around the time of their diagnosis. Employing a qualitative research design has enabled us to provide a greater depth of insight than could have been achieved using other methodologies, such as questionnaire designs [19, 23, 40]. Our study was further strengthened by having had three people involved in reviewing interview transcripts and undertaking data analysis; this was especially important as the first author (CW) had first-hand experience of TN which might, potentially, have biased his interpretation of the data. By being sensitive to lived experiences of people with TN, our study design permitted the perspectives of a marginalised, and, at times quite literally, voiceless patient group to be heard. However, by virtue of undertaking interviews by phone rather than face-to-face it possible that some non-verbal cues might have been missed. In addition, as a result of recruiting via online forums rather than through healthcare settings, it is possible that we interviewed an atypical patient group; indeed, because of the way we recruited it was impossible for us to determine how many people (and who) might have seen the study advertisement and chosen not to opt in. It was also not possible for us to formally confirm participants’ TN diagnoses as a result of our recruitment approach. As well as the sample being skewed towards White, educated individuals, study participants may have been motivated to take part as a result of having had more negative healthcare experiences. To address the study limitations, future research could focus upon Black, ethnic minority and other seldom heard groups. Additionally, to help improve diagnostic pathways and information provisioning, future research could usefully explore primary care practitioners’ perspectives together with those of General Practice Speciality trainers. Additional insights might also usefully be gained from interviewing significant others as these people might have an important role to play in helping individuals to seek and attain a timely TN diagnosis.

Given that TN is such a rare of the condition, it would have been extremely challenging to recruit sufficient numbers of recently diagnosed individuals. We therefore recruited participants regardless of when they first found out they had TN. While this meant we were successful in attaining data saturation, it is possible that some accounts may have been subject to recall bias. Although some participant accounts were historical, we would argue that our findings and recommendations continue to be extremely relevant as, to the best of our knowledge, little has changed in the clinical landscape in recent years to improve clinician’s training, knowledge and understanding of TN. Indeed, this is reflected in several recent publications where clinical experts have continued to emphasise the on-going challenges of diagnosing TN in a timely manner, particularly in primary care settings [8, 41].

## Conclusions

Study findings suggest that TN patients’ needs are poorly met both prior to and around the time of diagnosis. As well as helping to understand the detrimental impacts a delayed diagnosis can have on an individual’s health and wellbeing, our study highlights the importance of developing bespoke training for primary care professionals. Such training should not only serve to facilitate timely recognition of TN symptomatology but also help ensure that primary care professionals deliver a TN diagnosis in clear, sensitive and empathetic ways.

## Data Availability

The datasets generated and analysed in the course of this study are not publicly available due to risks to individual privacy.

## Appendix

**Table:** Topic guide

Can you tell me a bit about yourself e.g., Who you are? What you do? Who you live with?
Age, occupation (or previous occupation), education
Did you have any prior knowledge of TN before you were diagnosed?
Can you tell me a bit your experiences of being diagnosed?
What were the events leading up to the diagnosis?
When did you suspect there was a problem? What was your pain like at the time? What did you think was initially causing your pain? Did this influence what you did next?
How long did you wait before going to see a healthcare professional (why)?
Who did you present to? What happened next? What were you told? Did you feel you were being taken seriously at the time?
If a TN diagnosis didn’t happen, how did you feel, what did you do next?
Did you seek any information for non-medical sources (e.g., internet) prior to being diagnosed?
When and how did your diagnosis happen?
Who give you your diagnosis? What did they tell you at the time?
What were your thoughts and feelings at the time?
How confident did you feel in your diagnosis?
How did other people around you (partner/family/friends/colleagues) react?
What information and support were you given at the time your found out you had TN?
Did you feel you were given sufficient information about TN and how you should manage it?
How (at all) could the information and support given to people newly diagnosed with TN be improved?
